# New Zealand blackcurrant extract modulates the heat shock response in men during exercise in hot ambient conditions

**DOI:** 10.1007/s00421-024-05439-w

**Published:** 2024-03-07

**Authors:** Nathan J. Conrad, Emerson P. Heckler, Ben J. Lee, Garrett W. Hill, Tessa R. Flood, Lucy E. V. Wheeler, Rianne Costello, Ella F. Walker, Trevor L. Gillum, Mark E. T. Willems, Matthew R. Kuennen

**Affiliations:** 1 Department of Health & Human Performance, High Point University, One University Parkway, High Point, NC 27268 USA; 2 Occupational and Environmental Physiology Group, Centre for Physical Activity, Sport and Exercise Science, Coventry University, Coventry, England; 3 Institute of Sport, Manchester Metropolitan University, Manchester, UK; 4 Institute of Applied Sciences, University of Chichester, Chichester, UK; 5 Global Food Security Programme, Biotechnology and Biological Sciences Research Council, Swindon, UK; 6 Defence Science and Technology Laboratory, Porton Down, Salisbury, UK; 7 Department of Kinesiology, California Baptist University, Riverside, CA USA

**Keywords:** Exercise, Hyperthermia, Heat shock protein, Supplements, Anthocyanins

## Abstract

**Purpose:**

To determine if 7d of New Zealand blackcurrant (NZBC) extract alters the heat shock, inflammatory and apoptotic response during prolonged exertional-heat stress.

**Methods:**

Ten men (Age: 29 ± 2 years, Stature: 1.82 ± 0.02 m, Mass: 80.3 ± 2.7 kg, *V̇*O_2max_: 56 ± 2 mL·kg^−1^·min^−1^) ingested two capsules of CurraNZ^™^ (NZBC extract: 210 mg anthocyanins·day^−1^) or PLACEBO for 7d prior to 1 h treadmill run (65% *V̇*O_2max_) in hot ambient conditions (34 °C/40% RH). Blood samples were collected before (Pre), immediately after (Post), 1 h after (1-Post), and 4 h after (4-Post) exercise. Heat shock proteins (HSP90, HSP70, HSP32) were measured in plasma. HSP and protein markers of inflammatory capacity (TLR4, NF-κB) and apoptosis (BAX/BCL-2, Caspase 9) were measured in peripheral blood mononuclear cells (PBMC).

**Results:**

eHSP32 was elevated at baseline in NZBC(+ 31%; *p* < 0.001). In PLACEBO HSP32 content in PBMC was elevated at 4-Post(+ 98%; *p* = 0.002), whereas in NZBC it fell at Post(− 45%; *p* = 0.030) and 1-Post(− 48%; *p* = 0.026). eHSP70 was increased at Post in PLACEBO(+ 55.6%, *p* = 0.001) and NZBC (+ 50.7%, *p* = 0.010). eHSP90 was increased at Post(+ 77.9%, *p* < 0.001) and 1-Post(+ 73.2%, *p* < 0.001) in PLACEBO, with similar increases being shown in NZBC **(**+ 49.0%, *p* = 0.006 and + 66.2%, *p* = 0.001; respectively). TLR4 and NF-κB were both elevated in NZBC at PRE(+ 54%, *p* = 0.003 and + 57%, *p* = 0.004; respectively). Main effects of study condition were also shown for BAX/BCL-2(*p* = 0.025) and Caspase 9 (*p* = 0.043); both were higher in NZBC.

**Conclusion:**

7d of NZBC extract supplementation increased eHSP32 and PBMC HSP32 content. It also increased inflammatory and apoptotic markers in PBMC, suggesting that NZBC supports the putative inflammatory response that accompanies exertional-heat stress.

**Supplementary Information:**

The online version contains supplementary material available at 10.1007/s00421-024-05439-w.

## Introduction

Exercise in hot environments confers direct (thermal) and indirect (splanchnic vasoconstriction) stress on the gastrointestinal mucosa (Rowell 1974), altering the phosphorylation status of tight junction proteins and allowing lipopolysaccharide (LPS) to enter the blood circulation (Zuhl et al. 2014). There LPS binds Toll-like Receptor-4 (TLR4) on surveilling leukocytes, activating the nuclear factor kappa B (NF-κB) cascade and resulting in the production of pro-inflammatory cytokines [tumor necrosis factor-α (TNF-α), Interleukin-1-β (IL-1β), and Interleukin-6 (IL-6)] (Kuennen et al. 2011). Overactivation of this pathway (e.g., systemic inflammatory response syndrome) confers the greater core temperature rise, intravascular coagulation, and multiple organ failure that characterize exertional-heat stroke (EHS) (Lim 2018).

Given the preeminent role of the gastrointestinal tract in the etiology of exertional heatstroke, our group, and others have screened a variety of dietary supplements for protection against gastrointestinal barrier damage during exertional-heat stress. Improvements have been shown following short-term dietary supplementation with curcumin (Szymanski et al. 2018) and glutamine (Zuhl et al. 2015; Zuhl et al. 2014), but not probiotics (Gill et al. 2016), quercetin (Kuennen et al. 2011) or bovine colostrum (McKenna et al. 2017; Morrison et al. 2014). Recently, we reported reductions in gastrointestinal barrier permeability and enterocyte damage following one week of blackcurrant extract supplementation (equivalent to 210 mg of anthocyanins per day; for reference anthocyanins are a class of water-soluble flavonoids that promote antioxidant and anti-inflammatory functions) (Lee et al. 2022). Our interest in this supplement was predicated on data from animal models that reported improvements in serum antioxidant capacity and gut microbiota function (Jurgonski et al. 2014) in addition to other biomarkers of intestinal health (Paturi et al. 2018). Fecal microbiota evidence in humans indicates improved bacterial growth and inactivation of toxic bacterial enzymes following blackcurrant supplementation (Molan et al. 2014). Multiple preclinical trials with another anthocyanin-containing supplement (bilberry) have reported reductions in NF-κB mediated inflammatory cascades in ulcerative colitis patients (Biedermann et al. 2013; Roth et al. 2016).

Despite the improvements in gut barrier integrity (Intestinal Fatty-Acid Binding Protein; I-FABP) and function (lactulose/rhamnose ratio) that were shown following 1wk of NZBC-extract supplementation in our prior work (Lee et al. 2022), changes were not detected in markers of microbial translocation [Lipopolysaccharide Binding Protein (LBP) and Plasma-Soluble Cluster of Differentiation 14 (sCD14)]. Circulating cytokines that are associated with EHS risk (IL-1RA, IL-6, IL-10) were also not improved. Because heat shock proteins (HSP) intersect the otherwise linear relationship between gut barrier integrity and microbial translocation (and impart influence on pro-inflammatory cytokine cascades and oxidative-stress markers), in the present study we measured extracellular HSP (eHSP90, eHSP70, eHSP32) and intracellular HSP (HSP90, HSP70, HSP60, & HSP32) derived from peripheral blood mononuclear cell (PBMC) samples. In our prior work, we have shown the combination of these four HSP to be a thorough means of analyzing the heat shock response and its’ influence on pro- and anti-inflammatory cytokine cascades (Kuennen et al. 2011; Falgiano et al. 2018; Szymanski et al. 2018; Hill et al. 2020; Hill et al. 2020). Given the advent of newer technologies and our interest in inflammatory function (intracellular HSPs reduce pro-inflammatory cytokine cascades, whereas extracellular HSPs are typically pro-inflammatory), in the present study both extracellular and intracellular HSPs were examined. In addition, potential changes in PBMC-associated inflammatory cascades were assessed via measurement of TLR4, myeloid differentiation protein 88 (MyD88), phosphorylated I-κB-α (p-IκB-α), and NF-κB. Alterations in inflammatory status trigger changes in apoptotic markers in PBMC (Shen et al. 2019). For that reason, we also measured common markers of pro- and anti-apoptotic function in PBMC [B-cell lymphoma protein (BCL-2), BCL-2 associated X, apoptosis regulator (BAX), and Caspase 9].

## Methods

### Participants

Ten recreationally active men (Age: 29 ± 2 years, Stature: 1.82 ± 0.02 m, Mass: 80.3 ± 2.7 kg, VO_2max_: 56 ± 2 mL·kg^−1^·min^−1^) provided written informed consent prior to completing a double-blind placebo-controlled study with randomized, cross over design. The study was approved by the University of Chichester Research Ethics Committee with protocols and procedures conforming to the 2013 Declaration of Helsinki. All participants were non-smokers and negative for cardiovascular, pulmonary, or metabolic disease as defined by the American College of Sports Medicine (Riebe et al. 2015). These data are part of a larger data set, from which information on gastrointestinal barrier permeability, enterocyte damage, and circulating cytokine concentrations have been reported previously (Lee et al. 2022).

### Experimental design

Each participant completed one maximal exercise test, one familiarization trial, and two experimental trials. Participants were instructed to abstain from strenuous exercise and alcohol for 48 h prior, and caffeine-containing products on the day of testing. They were also asked to adhere to their normal exercise training schedule. All testing was conducted in the morning following a 12-h overnight fast. On the first visit, each participant completed an incremental exercise test to exhaustion on a motorized treadmill (HP Cosmos, Pulsar, h/p/cosmos Sports & Medical gmbh, Germany) to determine their maximal aerobic capacity (*V̇*O_2_max). They also completed a second exercise bout on that date to verify the workload that was required to elicit 65% of individual *V̇*O_2max_. The second visit was a FAM trial that familiarized participants with all measurements and study procedures under thermoneutral conditions (18 °C, 40% RH). This included 1 h of treadmill exercise at a workload equivalent to 65% *V̇*O_2_max (e.g., the same workload that was completed during their heat stress trials (e.g., Study Visits 3 and 4), which were separated by a minimum of 2 weeks to minimize the likelihood that carry-over effects influenced primary study outcomes.

### Dietary supplementation and diet monitoring

Participants used standard food diaries to record their dietary intake for 48 h prior to their first experimental visit. Upon completion of their first experimental visit, participants were given a copy of their food log and asked to replicate this dietary intake for all subsequent visits. Dietary intake was quantified using Nutritics software (Nutritics Ltd, Dublin, Ireland). Participants also completed a food frequency questionnaire that listed the amount and frequency of anthocyanin-containing foods and drinks to estimate their habitual anthocyanin intake (Neveu et al. 2010). Habitual anthocyanin intake from the diet was shown to be low, as reported previously (Lee et al. 2022). Prior to Visits 3 and 4, participants consumed 2 capsules of concentrated NZBC extract (2 × 300 mg active cassis containing 210 mg of anthocyanins, i.e., 35–50% delphinidin-3-rutinoside, 5–20% delphinidin-3-glucoside, 30–45% cyanidin-3-rutinoside, 3–10% cyanidin-3-glucoside per capsule; CurraNZ^™^, Health Currancy Ltd., Surrey, UK) or 2 capsules of identical-looking placebo capsules (2 × 300 mg microcrystalline cellulose M102) every morning for 7 days (Cook et al. 2015, 2017). One experimenter (M.E.T.W) made up visually identical NZBC and placebo pill packets for each participant, then left them in bulk in the principal investigator’s office (B.J.L) without any personal interaction. Blinding was not broken until after the sample analysis was completed. The 2 experimental conditions (NZBC and PLACEBO) were separated by a 14-day washout period. Trial order was determined using a free online tool (https://www.randomizer.org) and five participants received NZBC extract as the first condition. All experimental trials were conducted in an environmental chamber (TISS Services UK, Medstead, Hampshire, UK) controlled at 34.1 ± 0.1 °C and 40.8 ± 0.2% RH.

### Experimental protocol

On the morning of each experimental trial, participants were instructed to drink ~ 400 ml of water and arrived at the laboratory between 06:30 and 08:30. At that time a urine sample was taken for assessment of urine osmolality (Osmocheck PAL-OSMO; Vitech Scientific, Partridge Green, West Sussex, UK) and specific gravity (ATAGO 2791, ATAGO, Tokyo, Japan) to ensure participants were euhydrated (mOsmol^−1^ ≤ 600; USG ≤ 1.020) (Sawka et al. 2007). Following the recording of nude-body mass, participants inserted a polyethylene thermistor (Edale Instruments, Cambridge, UK) 10 cm past the external anal sphincter and were fitted with a heart rate monitor. Skin thermistors (Edale Instruments, Cambridge, UK) were attached to the mid-belly of the pectoralis major, triceps brachii, rectus femoris, and gastrocnemius for calculation of mean skin temperature (Ramanathan 1964). Standard equations were used to calculate mean body temperature and physiological strain index (PSI) during exercise (Kenney 1998; Moran et al. 1998).

**Fig. 1:**
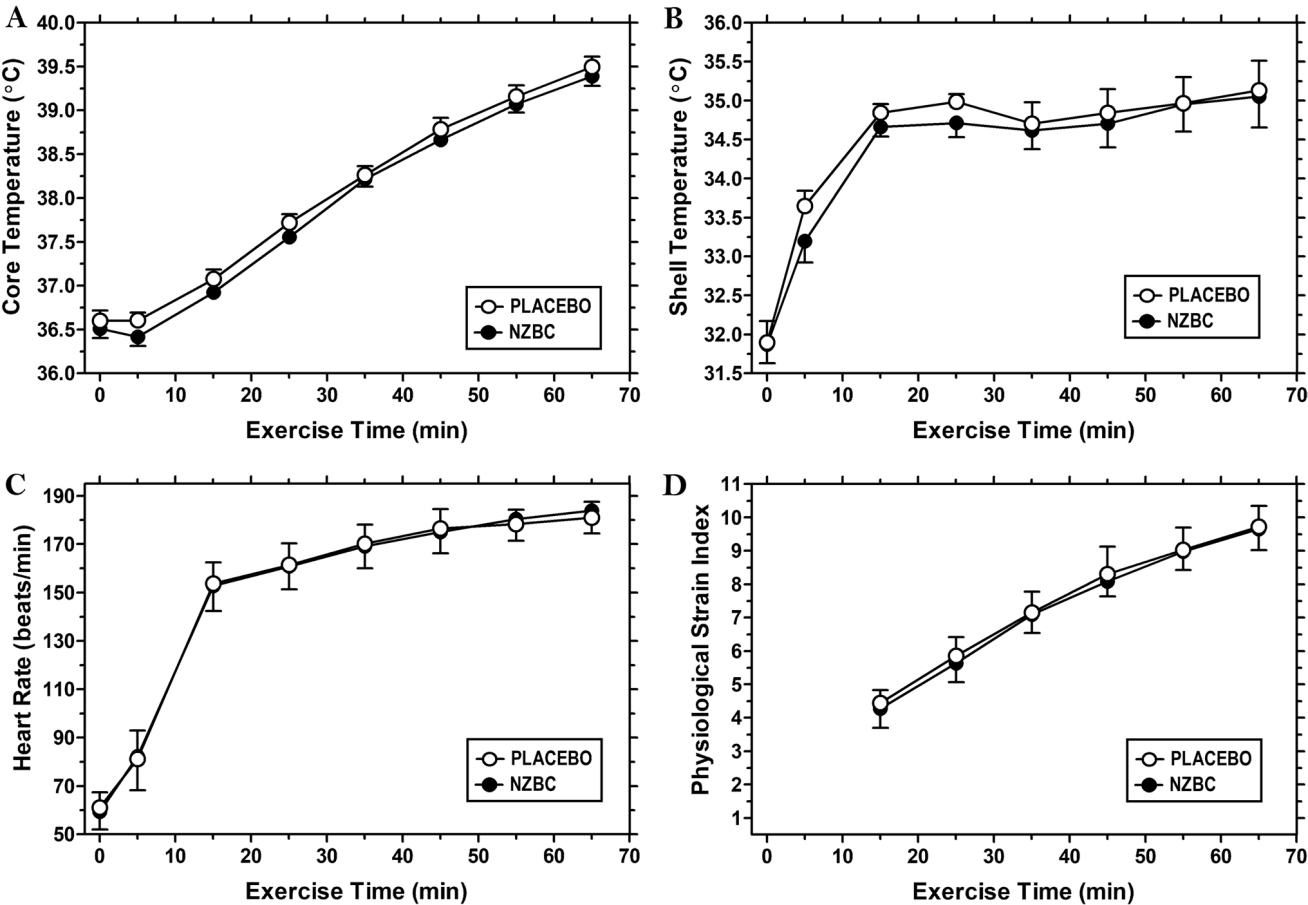
Physiologic stress response. Heart rate (**A**), rectal temperature (**B**), mean skin temperature (**C**), heart rate (**D**) and physiological strain index at rest and during the 60-min steady state run

Participants next rested in a supine position for 20-min, after which baseline physiologic measurements (HR, skin and rectal temperatures) were recorded. Participants next entered the environmental chamber where they rested for 5 min prior to 60 min of treadmill running at 65% *V̇*O_2max_ (1% incline). For reference, in Fig. 1 the measurement following 20-min of seated rest aligns to 0-min and the resting measurement taken inside the environmental chamber aligns to the 5-min mark. Following these two resting measurements, heart rate, rectal and skin temperatures were recorded at 10-min intervals throughout exercise. Bottled water (chamber temperature) was available ad libitum during each trial, and the volume of ingested fluid was recorded. On completion of exercise, participants towel-dried and nude-body mass was reassessed. The difference in pre-to-post-exercise body mass was used to calculate sweat rate (corrected for water ingestion but not respiratory water loss).

### Blood collection

Before exercise (Pre), within 20 min following exercise cessation (Post), 1 h after exercise (1-Post), and 4 h after exercise (4-Post) blood samples were drawn from an antecubital vein using standard venipuncture techniques. From these blood samples, extracellular HSP were measured at Pre, Post, and 1-Post using standard ELISA kits (Proteintech, Manchester, UK). Peripheral blood mononuclear cells (PBMC) were isolated from 5 ml EDTA blood that had been layered upon 3 ml Ficoll (10,771, Sigma Aldrich, Gillingham, UK) inside of Leucosep tubes (163290P, Greiner Bio-One, Stonehouse, UK) prior to centrifugation at 800 g for 20 min. Isolated PBMC were washed thrice in 10 ml PBS at 400 g for 10 min. Clean PBMC were transferred into sterile 1.7 ml micro-eppendorf tubes and frozen at − 80 °C until later batch analysis (described below).

### Immunoblotting and protein expression

PBMC were lysed in RIPA buffer from Bio-Rad (Hercules, CA, USA) supplemented with protease inhibitor mix (0.1%) from Bio-Rad (Hercules, CA, USA) on ice for 1 h. Insoluble material was removed, and protein concentrations were determined by Bradford assay from Bio-Rad (Hercules, CA, USA). 50 μg of total protein from each sample was size-separated on an 8–10% sodium dodecyl sulfate–polyacrylamide gel using electrophoresis, after which proteins were electro-transferred to PVDF membranes. Membranes were blocked in TBST-5% non-fat milk powder for > 90 min, after which they were probed at 4ºC overnight with target antibodies from Santa Cruz Biotechnologies (Santa Cruz, CA, USA) or Abcam (Cambridge, MA, USA) or Enzo Life Sciences (Farmingdale, NY, USA), as indicated in Supplemental Table 1. Unless stated otherwise, protein loading was confirmed with anti-β-Actin primary antibody from Santa Cruz Biotechnologies (Santa Cruz, CA, USA) at a dilution of 1:400 in TBST-5% non-fat milk powder. Bound antibodies were detected by horseradish peroxidase-conjugated secondary antibodies from Santa Cruz Biotechnologies (Santa Cruz, CA, USA) or Abcam (Cambridge, MA, USA) at a dilution of 1:5000 in TBST-5% non-fat milk powder for 1 h at room temperature on an orbital platform at 250RPM. Protein signal intensities were determined by chemiluminescence using the Clarity Western ECL substrate kit from Bio-Rad (Hercules, CA, USA) and imaged using the ChemiDoc Touch from Bio-Rad (Hercules, CA, USA). Relative signal intensities were normalized to β-Actin and quantified using Image Lab from Bio-Rad (Hercules, CA, USA). A complete list of all primary antibodies that were utilized to generate Western Blots in this experiment is provided in Supplemental Table 1.

### Statistical analysis

Statistical analyses were performed using STATISTICA for Windows (version 7.1; StatSoft Inc., Tulsa, OK, USA). Unless stated otherwise, text and table data are presented as mean ± SEM for *N* = 10. As reported previously (Lee et al. 2022), two-tailed paired t tests were used to determine if dietary intake, urine specific gravity, water ingestion, and sweat rate were different between conditions (PLACEBO or NZBC). Two-factor RM-ANOVAs [where study condition (PLACEBO or NZBC) and exercise time (0–60 min) served as the repeated measures factors] were used to examine potential differences in core and shell temperatures, HR, and PSI over the 60 min exercise bout. Statistical analysis of extracellular HSP were conducted on the absolute concentrations, after correction for plasma volume change. Western blot data were analyzed with two-factor RM-ANOVAs, where intervention (PLACEBO or NZBC) and blood sample timepoint (Pre, Post, 1-Post, 4-Post) served as the repeated measures factors. Statistical significance was set at *p* ≤ 0.05. Significant main and interaction effects were further evaluated by way of Duncan’s New Multiple Range Test. To ensure clear articulation of study findings, the two study conditions (PLACEBO and NZBC) were also separated and one-way RM-ANOVAs were run on each (to examine the main effect of time), followed by Duncan’s New Multiple Range Test (where appropriate). Effect sizes were calculated for all significant main and interaction effects [partial eta squared (*η*_p_^2^)]. For reference, *η*_p_^2^ values of 0.01, 0.09, and 0.25 are considered to be small, medium, and large effect sizes; respectively (Cohen 1992).

### Power analysis

A power analysis was conducted (G*PowerVersion 3.1; Dusseldorf, North Rhine-Westphalia, Germany) using the means and SDs of a prior research study that examined the effect of short-term dietary quercetin supplementation on exertional-heat stress-mediated changes in protein expression in PBMC (Kuennen et al. 2011). It was determined that with an α-level of *p* ≤ 0.05, eight participants would result in a 92% probability (i.e., 1–*β*) of detecting differences in protein expression in PBMC.

## Results

### Cardiovascular and thermoregulatory responses

Cardiovascular and thermoregulatory responses were not different between study conditions (all interaction effects *p* > 0.05). Significant main effects (of time) indicate exercise conferred elevations in heart rate (*F* = 796.90, *p* < 0.0001, *η*_p_^2^ = 0.989) (Fig. 1A), rectal temperature (*F* = 387.40, *p* < 0.0001, *η*_p_^2^ = 0.977) (Fig. 1B), skin temperature (*F* = 37.86, *p* < 0.0001, *η*_p_^2^ = 0.808) (Fig. 1C), and physiological strain (*F* = 345.40, *p* < 0.0001, *η*_p_^2^ = 0.975) (Fig. 1D). As reported previously (Lee et al. 2022), fluid intake and whole-body sweat rate were also not different between study conditions (*p* = 0.938 and 0.465; respectively).

### eHSP32

**Fig. 2:**
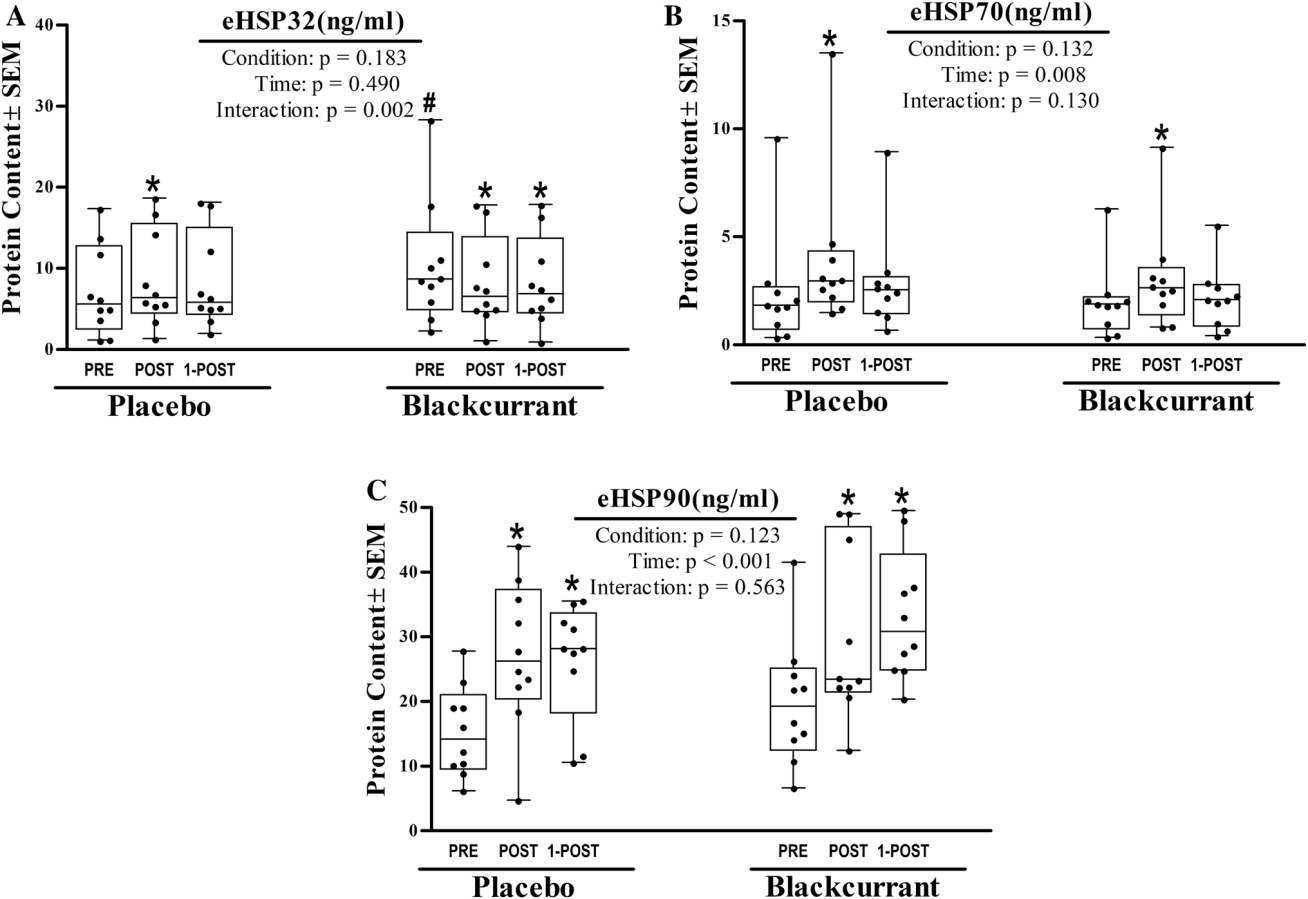
Extracellular heat shock response. Impact of 7d of New Zealand blackcurrant (NZBC) extract supplementation (equivalent to 210 mg anthocyanin·day^−1^) on extracellular concentrations of (**A**) eHSP32, (**B**) eHSP70, and (**C**) eHSP90. eHSP were assayed from plasma samples collected before (PRE), after (POST), and 1 h after (1-POST), and 4 h after 60 min of submaximal treadmill exercise (workload) performed under hot (37 °C), dry (25% RH) ambient conditions. Data for subjects (*N* = 10) are presented as box plots that display individual data points (closed circles), the 25 and 75th interquartile ranges (boxes), and the median (mid-line). Whiskers illustrate the highest and lowest value at each timepoint. Data were analyzed with two-factor RM-ANOVAs, where intervention (PLACEBO or NZBC) and blood sample timepoint (Pre, Post, 1-POST) served as the repeated measures factors. Statistical significance was set at *p* ≤ 0.05. Significant main effects and interactions were further evaluated using Duncan’s post hocs. #Significant between condition effect. *Significant within condition effect

The interaction effect for eHSP32 was significant (*F* = 9.445, *p* = 0.002, *η*_p_^2^ = 0.512) (Fig. 2A). *Post-hoc* analysis indicated that eHSP32 was elevated at baseline in NZBC as compared to PLACEBO baseline expression (+ 31.3%; *p* < 0.001). When conditions were run separately the main effect of time was significant in PLACEBO (*F* = 3.775, *p* = 0.043, *η*_p_^2^ = 0.295), where eHSP32 was shown elevated above baseline at Post (+ 20.1%; *p* = 0.021). While the main effect of time was also significant in NZBC (*F* = 5.287, *p* = 0.016, *η*_p_^2^ = 0.370), in this condition eHSP32 was shown elevated at baseline before falling below Pre-exercise values at Post (− 21.9%; *p* = 0.014) and 1-Post (− 21.5%; *p* = 0.012) exercise.

### eHSP70

The interaction effect for eHSP70 was not significant (*F* = 2.290, *p* = 0.130, *η*_p_^2 ^= 0.203) (Fig. 2B). A significant main effect (of time) was shown in both PLACEBO (*F* = 8.374, *p* = 0.003, *η*_p_^2^ = 0.482) and NZBC (*F* = 4.731, p = 0.022, *η*_p_^2 ^= 0.345). eHSP70 increased at Post in PLACEBO (+ 55.6%; *p* = 0.001) and at Post in NZBC (+ 50.7%; *p* = 0.010).

### eHSP90α

The interaction effect for eHSP90α was not significant (*F* = 0.593, *p* = 0.563, *η*_p_^2^ = 0.062) (Fig. 2C). A significant main effect (of time) was shown in both PLACEBO (*F* = 21.292, *p* < 0.001, *η*_p_^2 ^= 0.703) and NZBC (*F* = 9.786, *p* = 0.001, *η*_p_^2 ^= 0.521). eHSP90α increased at Post (+ 77.9%; *p* < 0.001) and 1-Post (73.2%; *p* < 0.001) exercise in PLACEBO. It also increased at Post (+ 49.0%; *p* = 0.006) and 1-Post (66.2%; *p* = 0.001) exercise in NZBC.

#### HSP32

**Fig. 3:**
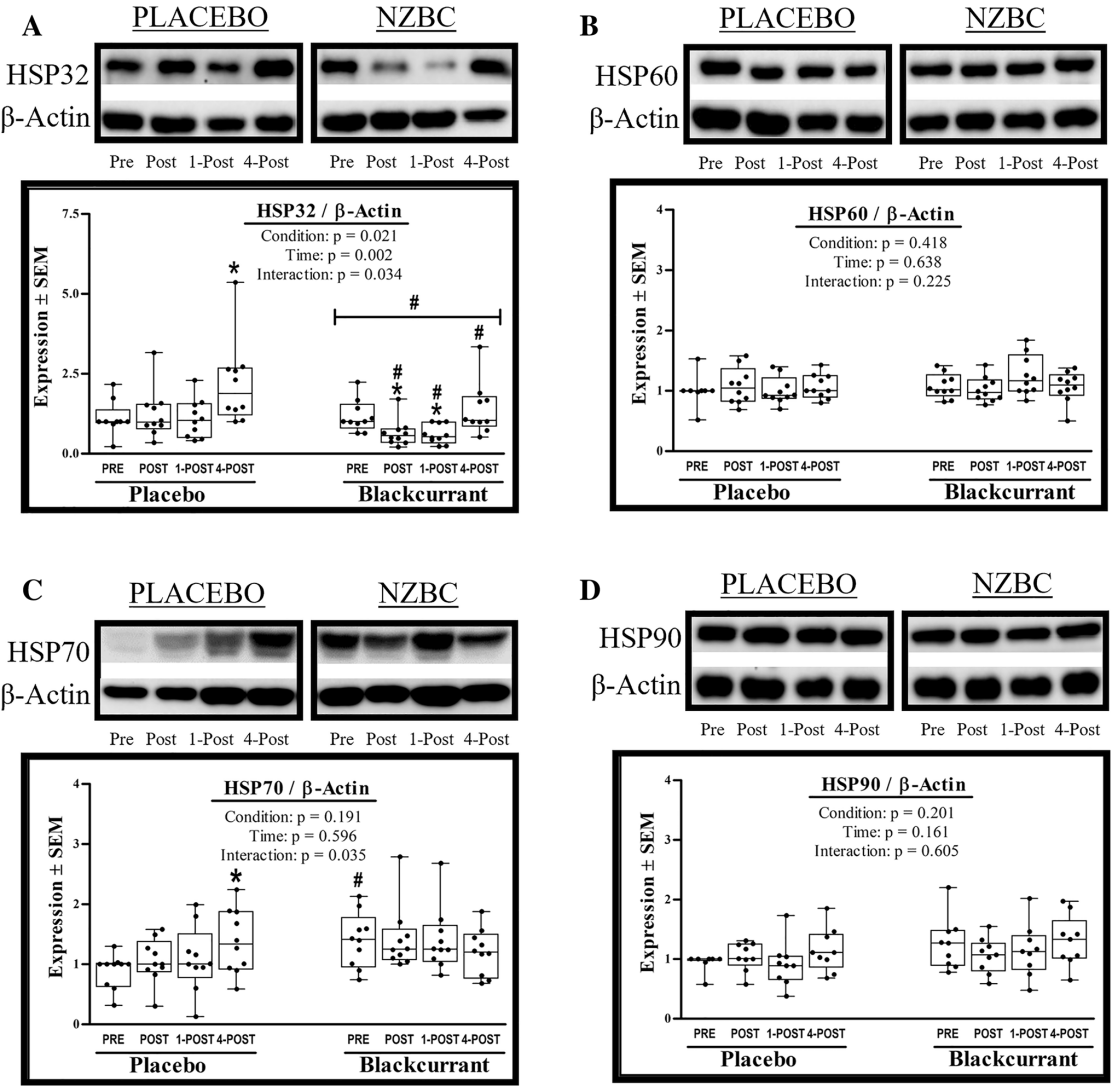
Intracellular heat shock tesponse. Impact of 7d of New Zealand blackcurrant (NZBC) extract supplementation (equivalent to 210 mg anthocyanin·day^−1^) on intracellular concentrations of (**A**) HSP32, (**B**) HSP60, (**C**) HSP70, and (**D**) HSP90. Changes in protein expression were determined in peripheral blood mononuclear cells (PBMC), which were isolated from plasma samples that were collected before (PRE), after (POST), 1 h after (1-POST), and 4 h after (4-POST) 60 min of submaximal treadmill exercise (workload) performed under hot (37 °C), dry (25% RH) ambient conditions. Data for subjects (*N* = 10) are presented as box plots that display individual data points (closed circles), the 25 and 75th interquartile ranges (boxes), and the median (mid-line). Whiskers illustrate the highest and lowest value at each timepoint. Data were analyzed with two-factor RM-ANOVAs, where intervention (PLACEBO or NZBC) and blood sample timepoint (Pre, Post, 1-POST, 4-POST) served as the repeated measures factors. Statistical significance was set at *p* ≤ 0.05. Significant main effects and interactions were further evaluated using Duncan’s post hocs. #Significant between condition effect. *Significant within condition effect

The interaction effect for HSP32 was significant (*F* = 3.339, *p* = 0.034, *η*_p_^2^ = 0.271) (Fig. 3A). *Post-hoc* analysis indicated HSP32 levels in PLACEBO and NZBC differed at Post (*p* = 0.012), 1-Post (*p* = 0.029), and 4-Post exercise (*p* = 0.001). Interestingly, in PLACEBO HSP32 was elevated at Post (+ 12.8%) and markedly elevated at 4-Post (+ 98.2%; *p* = 0.002). Whereas in NZBC, HSP32 fell at Post (-44.8%) and 1-Post (-48.2%) exercise before returning to baseline values at 4-Post.

#### HSP60

The interaction effect for HSP60 was not significant (*F* = 1.549, *p* = 0.225, *η*_p_^2^ = 0.147) (Fig. 3B). The main effects of condition and time were not significant, and there were also no main effects of exercise time when the two conditions were analyzed separately (all *p* > 0.05).

#### HSP70

The interaction effect for HSP70 was significant (*F* = 3.322, *p* = 0.035, *η*_p_^2^ = 0.270) (Fig. 3C). *Post-hoc* analysis indicated that HSP70 was elevated at baseline in NZBC as compared to PLACEBO baseline expression (+ 40%; *p* = 0.010). Although HSP70 was also visually elevated at POST and 1-POST exercise in NZBC, these levels did not achieve statistical significance (*p* = 0.062 and 0.090; respectively). When conditions were run separately there was a significant main effect (of time) in PLACEBO (*F* = 3.382, *p* = 0.033, *η*_p_^2^ = 0.273), wherein HSP70 increased above baseline levels at 4-Post (+ 54.9%; *p* = 0.007).

#### HSP90

The interaction effect for HSP90 was not significant (*F* = 0.796, *p* = 0.508, *η*_p_^2^ = 0.091) (Fig. 3D). The main effects of condition and time were not significant, and there were also no main effects of exercise time when the two conditions were analyzed separately (all *p* > 0.05).

#### TLR4

**Fig. 4:**
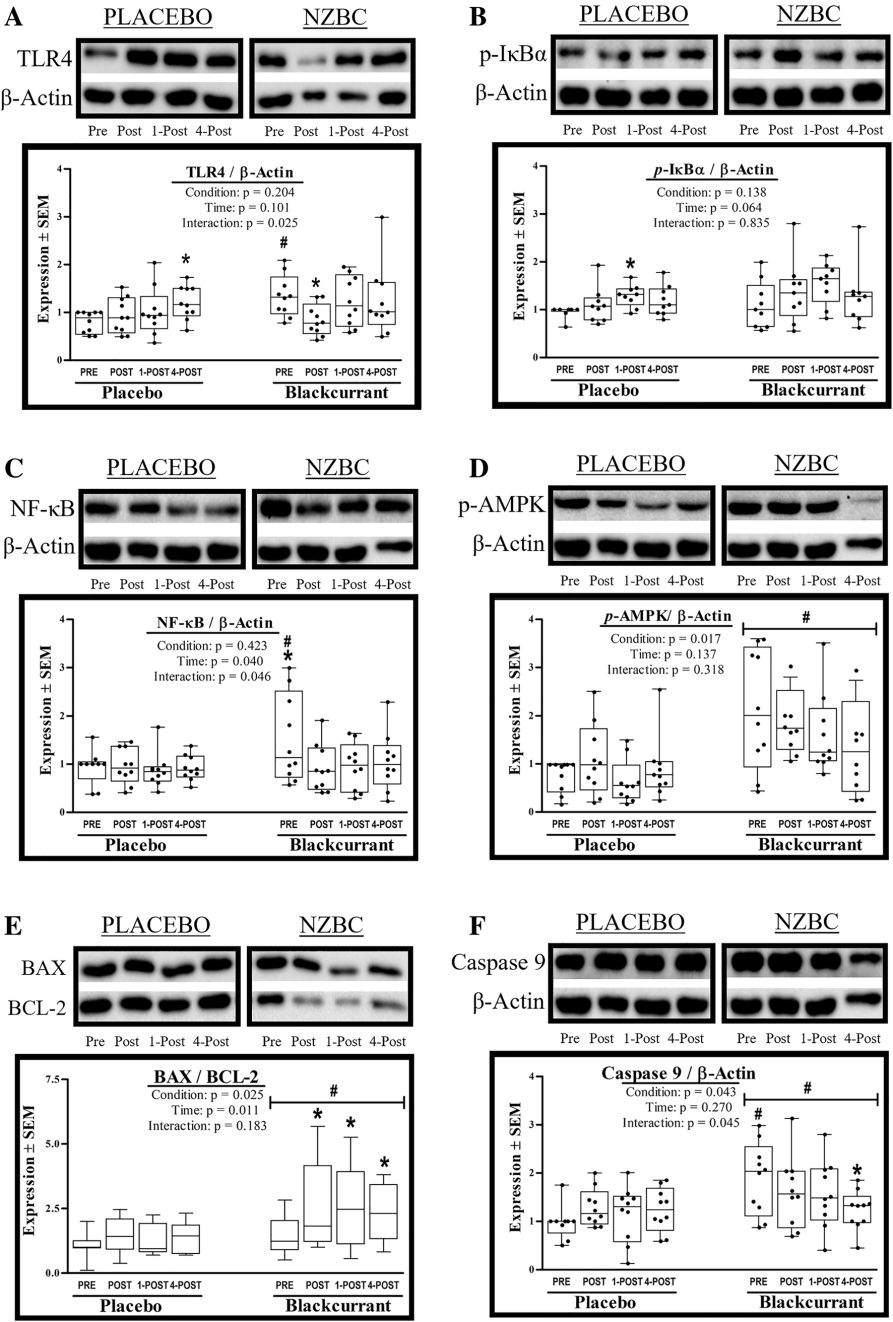
Inflammation and apoptosis. Impact of 7d of New Zealand blackcurrant (NZBC) extract supplementation (equivalent to 210 mg anthocyanin·day^−1^) on intracellular concentrations of (**A**) TLR4 (**B**) p-IκBa, (**C**) NF-κB, (**D**) p-AMPK, (**E**) BAX/BCL-2, and (**F**) Caspase 9. Changes in protein expression were determined in peripheral blood mononuclear cells (PBMC), which were isolated from plasma samples that were collected before (PRE), after (POST), 1 h after (1-POST), and 4 h after (4-POST) 60 min of submaximal treadmill exercise (workload) performed under hot (37 °C), dry (25% RH) ambient conditions. Data for subjects (*N* = 10) are presented as box plots that display individual data points (closed circles), the 25 and 75th interquartile ranges (boxes), and the median (mid-line). Whiskers illustrate the highest and lowest value at each timepoint. Data were analyzed with two-factor RM-ANOVAs, where intervention (PLACEBO or NZBC) and blood sample timepoint (Pre, Post, 1-POST, 4-POST) served as the repeated measures factors. Statistical significance was set at *p* ≤ 0.05. Significant main effects and interactions were further evaluated using Duncan’s post hocs. #Significant between condition effect. *Significant within condition effect

The interaction effect for TLR4 was significant (*F* = 3.654, *p* = 0.025, *η*_p_^2^ = 0.289) (Fig. 4A). Post-hoc analysis indicated TLR4 was elevated at baseline in NZBC as compared to PLACEBO baseline expression (+ 54%; *p* = 0.003). Under PLACEBO TLR4 was shown increased above baseline at 4post (+ 50.8%, *p* = 0.017) whereas in NZBC TLR4 fell below baseline at Post (− 37.7%, *p* = 0.020) before returning to baseline values by 1-Post exercise.

### p-IκBa

Neither main effect nor the interaction effect were significant for p-IκBa (Fig. 4B). When conditions were run separately the main effect of time was significant in PLACEBO only (*F* = 3.163, *p* = 0.043, *η*_p_^2 ^= 0.283), wherein p-IκBa rose above baseline at 1-Post (+ 35.1%; *p* = 0.011).

### NF-κB

The interaction effect for NF-κB was significant (F = 3.087, p = 0.046, *η*_p_^2^ = 0.278) (Fig. 4C). Post-hoc analysis indicated NF-κB was elevated at baseline (+ 57%; *p* = 0.004) in NZBC. When conditions were run separately the main effect of time was significant in NZBC only (*F* = 3.904, *p* = 0.019, *η*_p_^2 ^= 0.303), wherein NZBC was shown to be reduced below baseline at Post (− 39.9%, *p* = 0.008), 1-Post (− 37.1%, *p* = 0.012), and 4-Post (− 30.4%, *p* = 0.028) exercise.

### p-AMPK

The main effect of condition was significant (*F* = 11.380, *p* = 0.008, *η*_p_^2^ = 0.558) for p-AMPK, reflecting ~ 100% increase in p-AMPK at all time points in NZBC (Fig. 4D). However, neither the main effect of time (*F* = 2.517, *p* = 0.079, *η*_p_^2^ = 0.219) nor the interaction effect (*F* = 1.577, *p* = 0.218, *η*_p_^2^ = 0.149) achieved statistical significance. When conditions were run separately the main effect (of time) was not significant in either study condition.

### BAX/BCL-2 ratio

The interaction effect for BAX/BCL-2 was not significant (*F* = 1.737, *p* = 0.183, *η*_p_^2^ = 0.162). However, there was a significant main effect of condition (*F* = 7.163, *p* = 0.025, *η*_p_^2 ^= 0.443) reflecting greater BAX/BCL-2 in NZBC as compared to PLACEBO (Fig. 4E). When conditions were run separately, the main effect of time was significant in NZBC only (*F* = 4.486, *p* = 0.011, *η*_p_^2 ^= 0.333), where BAX/BCL-2 was shown to be elevated above baseline at Post (+ 43.5%; *p* = 0.006), 1-Post (+ 46.2%; *p* = 0.006), and 4-Post (+ 31.3%; *p* = 0.020) exercise.

### Caspase 9

The main effect of condition was significant (*F* = 5.514, *p* = 0.043, *η*_p_^2 ^= 0.380), reflecting ~ 41% greater Caspase 9 in NZBC as compared to PLACEBO (Fig. 4F). The interaction effect was also significant (*F* = 3.064, *p* = 0.045, *η*_p_^2^ = 0.254), where post-hoc analysis reflected greater Caspase 9 content in NZBC at baseline (+ 90%; *p* = 0.001). It is worthwhile to point out that when conditions were run separately the main effect of time was only significant in the NZBC condition (*F* = 3.715, *p* = 0.023, *η*_p_^2 ^= 0.292), wherein Caspase 9 had fallen below baseline levels at 4-Post (− 34.7%; *p* = 0.004).

## Discussion

In our prior work, we reported decreased gut barrier permeability and damage following one week of NZBC-extract supplementation, but microbial translocation and circulating cytokine concentrations were not shown to be altered (Lee et al. 2022). Because HSP intersect the otherwise linear relationship between gut barrier integrity and microbial translocation via their influence on pro-inflammatory cytokine cascades, in the present study we examined extracellular (eHSP90, eHSP70, eHSP32) and intracellular HSP (HSP90, HSP70, HSP60, HSP32) that had been derived from PBMC samples. For reference, prior work has shown that human cells secrete relatively low levels of HSP under normal physiologic conditions (Ogura et al. 2008), but plasma levels are greatly increased following exercise and/or thermal stress exposure (Jay et al. 2022; Periard et al. 2012). Dietary supplements that promote gastrointestinal health, including Rhodiola rosea (Jafari et al. 2022) and probiotics (Gill et al. 2016), have not been shown to alter basal eHSP concentrations or influence eHSP responses following endurance exercise (Marshall et al. 2017; Shanely et al. 2014).

As such, the elevated eHSP32 and HSP32 that were shown following NZBC-extract supplementation in the present study were unexpected. However, because NZBC-extract supplementation was also shown to elevate TLR4 and NF-κB content, we postulate that elevated eHSP32 may help coordinate the inflammatory response in PBMCs under basal conditions (Asea et al. 2000; Jay et al. 2022). This hypothesis is supported by the elevated NF-κB and TLR4 that were shown in PBMC under basal conditions following NZBC supplementation. For reference, eHSP90 binds to TLR4 expressed on NK cells and monocytes, activating these leukocytes to increase NF-κB mediated production of pro-inflammatory cytokines and improve their cidal capacity (Zhang et al. 2017). Similar findings have been reported for eHSP32, which has been shown to be elevated in chronic inflammatory conditions such as diabetes mellitus (Bao et al. 2010) and hemophagocytic lymphohistiocytosis (Miyazaki et al. 2010).

While plasma concentrations of eHSP70 and eHSP90 were also shown to increase following exertional-heat stress, this increase was equivalent across PLACEBO and NZBC-extract conditions. To our knowledge this study is the first to examine eHSP90 in conjunction with exertional-heat stress, whereas eHSP70 release has been relatively well characterized as an intensity-dependent phenomenon (Periard et al. 2012). Given that prior work, the modest (~ 150%) increase in eHSP70 that was shown in the present study was likely due to our moderate exercise intensity (60-min treadmill at 65% *V̇*O_2max_), whereas long-duration exercise has been shown to provoke greater eHSP70 responses (~ 2200% in an ironman triathlon and ~ 1674% in an 100 km run) (Gomez-Merino et al. 2006).

Overall, the changes in intracellular HSP that were shown in the present study were more subdued than the changes that were reported in eHSP. Of these, changes in HSP32 were the most interesting, where PBMC HSP32 content following NZBC-extract supplementation was shown reduced at Post, 1-Post, and 4-Post exercise. This contrasted with the PLACEBO condition, where HSP32 had nearly doubled (+ 98%) by 4 h post-exercise. Reduced HSP32 content following anthocyanin-rich NZBC-extract supplementation was unexpected because anthocyanin’s have previously been shown to increase HSP32 content and promote anti-inflammatory and immune supportive effects (Funes et al. 2020). Other dietary supplements that exhibit strong antioxidant effects have also been shown to upregulate HSP32, protecting mice from kidney damage following thermal burn injury (Guo et al. 2021). However, reduced HSP32 is not unprecedented, as Chen et al. (2022) reported lower HSP32 in the skeletal muscle of exercising Kunming mice following supplementation with vitamin C and *L. plantarum KSFY01* (a probiotic with antioxidant effects) (Chen et al. 2022). Given that HSP32 helps maintain homeostasis during exertional-heat stress (Taylor et al. 2012) and also affords protection against ischemia–reperfusion injury (Zeynalov et al. 2009), we think a plausible explanation is that HSP32 was increased under PLACEBO conditions to combat the stress associated with reductions in gut barrier function because the lactulose/rhamnose ratio and I-FABP were both shown elevated following PLACEBO supplementation but not under NZBC-extract supplementation (Lee et al. 2022). Although they utilized a slightly different model than our own, Peng (2000) has also shown a reduced-HSP32 response in lymphocytes that were collected from human subjects following 5 weeks of antioxidant supplementation, then subjected to ex vivo heat shock (42.5 °C for 45 min) (Peng et al. 2000).

Prior reports have examined changes in HSP70 content following short-term dietary supplementation with probiotics and glutamine. The 45% increase in HSP70 expression in PBMCs following 1wk of NZBC-extract supplementation that was shown in the present study is similar to the 35% increase in HSP70 expression following a single dose (0.9 g/kg of fat-free mass) of dietary glutamine supplementation (Zuhl et al. 2015). Likely owing to the difference in cell/tissue type, 8 weeks of 500 mg/d vitamin C supplementation has been shown to elevate the HSP70 content of skeletal muscle ~ sixfold (Khassaf et al. 2003). Collectively, these data support greater basal activation of the heat shock response to promote enhanced physiologic function and greater stress tolerance. Interestingly in all three of these studies HSP70 did not increase further with exercise heat stress. Whereas HSP70 did increase with exercise under PLACEBO supplementation in the present study, suggesting thermal tolerance may have been lower under PLACEBO supplementation. Evidence has suggested that anthocyanin-rich supplements increase HSP70 induction in the GI tract of mice (Murakami et al. 2015) and there is also some corresponding evidence from anthocyanin-supplemented Caco-2 models that were subjected to severe levels of oxidative stress (Bei et al. 2008). A meta-analysis on heat acclimation-induced intracellular HSP70 in humans has also provided evidence supporting the relationship between the HSP70 content of PBMCs and corresponding changes in gastrointestinal barrier function (Nava and Zuhl 2020). In this context, the upregulation of basal HSP70 in PBMCs following NZBC supplementation may provide some indirect evidence of HSP induction in the gut, possibly explaining the improvements in the lactulose/rhamnose ratio and I-FABP responses that were shown in our prior work (Lee et al. 2022).

Extracellular HSPs are known to promote pro-inflammatory cascades that reciprocally inhibit apoptotic drive. In our prior report on NZBC extract and exertional-heat stress (Lee et al. 2022), we did not detect any changes in inflammatory cytokine production, so in the present study we wanted to determine if inflammatory markers were altered in PBMC. Interestingly, we found that TLR4 and NF-κB were both elevated at baseline under NZBC-extract supplementation. These changes did not elevate inflammatory signaling with exercise, as circulating cytokine concentrations were not different between NZBC and PLACEBO conditions (Lee et al. 2022), and PBMC NF-κB content actually fell Post-exercise. TLR4 content did rise with exercise, but only under PLACEBO conditions. As such, the mechanism to explain such changes remains unclear.

However, similar to what has been reported for eHSP70, recent work suggests that TLR4 may also be regulated by exercise intensity (Ducharme et al. 2024). In addition to membrane-bound protein levels, recent research has shown that assessment of circulating/soluble TLR4 may offer greater insight into TLR4 expression and signaling in response to exercise (Ducharme et al. 2024;Ducharme et al. 2023; Perkins et al. 2023). As soluble TLR4 has been shown to function as a decoy receptor to TLR4 ligands and thus reduced TLR4-mediated signaling (Ducharme et al. 2023; Iwami et al. 2000) it is possible that an increased concentration of both membrane-bound and soluble TLR4 may help to explain the absence of increased inflammatory signaling with exercise under NZBC. Acute exercise has been shown to change the fraction of PBMC subpopulations that express TLR4, which could potentially explain the increase in TLR4 expression with moderate exercise that was shown in the present study. For example, acute exercise has been shown to increase shear stress that can demarginalize NK cells, effectively increasing the number of circulating peripheral blood NK cells (Millard et al. 2013). As such, an increase in TLR4-expressing NK cells could be a plausible explanation for the rise in TLR4 expression that was shown under PLACEBO conditions in the present study, one that would not necessitate an increase in inflammatory cytokine production.

It is well established that high-intensity exercise increases intestinal permeability (Pals et al. 1997), leading to activation of the NF-κB pro-inflammatory pathway and transcription of pro-inflammatory cytokines (e.g., TNF-α, IL-1β, IL-6) (Selkirk et al. 2008). An in vitro study that explored the effect of anthocyanin-rich blackcurrant supplementation on THP-1 cells reported pre-incubation with NZBC inhibits NF-κB mediated pro-inflammatory cascades and reduces LPS-stimulated TNF-α by ~ 50% (Lyall et al. 2009). However, in that study, total NF-κB content was unchanged (Lyall et al. 2009). Another in vitro study reported that blackcurrant juice lowers NF-κB activity and LPS-mediated inflammation in cultured macrophages (Huebbe et al. 2012). The in vivo data from the present study do not agree with those prior in vitro findings, suggesting that differences in the blackcurrant-dosing strategy and reduced-NZBC anthocyanin bioavailability across human gastrointestinal tissues may help to explain these discordant findings.

BCL-2 and BAX are intracellular proteins that restrain and promote apoptosis, respectively (Shen et al. 2019). They play key roles in the maintenance of mitochondrial membrane function and cytochrome C release (Slee et al. 1999). After release of cytochrome C from the mitochondria, Caspase-9 binds to apoptotic peptidase activating factor-1 to form the apoptosome that leads to Caspase-9 activation and production of downstream executioner caspases like Caspase-3 and Caspase-7 (Slee et al. 1999). Researchers commonly use the ratio of BAX to BCL-2 (BAX:BCL-2) as an index of apoptotic drive and measure Caspase-9 to verify this outcome. In the present study, although BAX:BCL-2 was not different between study conditions at baseline, under NZBC BAX:BCL-2 was shown to rise at Post (+ 44%), 1-Post (+ 46%), and 4-Post (+ 31%) exercise. Caspase 9, which is one of the primary coordinators of apoptotic cell death, was also elevated at baseline in NZBC. When taken together, these data suggest PBMCs experienced a greater apoptotic drive under NZBC.

The literature on inflammatory cytokines and apoptosis is somewhat less clear, as they can be pro- or anti-apoptotic depending on the concentration and cell type. Crosstalk between cytokines adds further confusion. For example, IL-6 acts as an initiator to produce anti-inflammatory cytokines (IL-1RA and IL-10) (Steensberg et al. 2003) that help to offset elevated levels of other pro-inflammatory cytokines (TNF-α and IL-1β) (Schindler et al. 1990). Interestingly, multiple NF-*κ*B-dependent genes that are induced by TNF-*α* have been shown to protect cells against apoptosis (Baichwal and Baeuerle 1997). Likewise, elevations in HSP70 have been shown to inhibit heat-induced apoptosis via prevention of BAX translocation into the mitochondria (Stankiewicz et al. 2005). Antioxidant supplementation in animal heat-stroke models provides strong evidence that elevations in HSP70 content help to reduce NF-*κ*B-mediated signaling cascades and also result in less cleaved Caspase 3, which is the final downstream caspase prior to apoptotic cell death (Shen et al. 2019). In conjunction with HSP70, animal models also provide evidence that geldanamycin-mediated elevations in p-AMPK help to reduce apoptosis and defend against lethal heat stroke (Tsai et al. 2016). This is intriguing when one considers that p-AMPK and HSP70 were both shown to be upregulated at baseline following NZBC-extract supplementation, but failed to increase further in response to exertional-heat stress. As such, it is possible that the lack of increase in these mediators, in conjunction with the lack of increase in inflammatory cytokine production during exertional-heat stress under NZBC supplementation may have contributed to the elevated apoptotic drive we report here.

### Limitations

One limitation of this research is the focus on males. Future work should examine NZBC responses across both biologic sexes (men and women). It may also be worthwhile to examine NZBC in conjunction with other polyphenol substances (such as curcumin: Szymanski et al. 2018) and through the use of more novel investigative techniques (such as microRNA). In addition, it is worthwhile to note that a greater expression of TLR4 and NF-*κ*B at baseline under NZBC may suggest a greater percentage of TLR4 expressing cells within the PBMC fraction being sampled. Since the percentage of TLR4 expressing cells within the PBMC fraction were not evaluated in the present study, this represents an additional potential limitation of our manuscript.

## Conclusion

The present study provides evidence that 7d intake of anthocyanin-rich NZBC increases extracellular and intracellular HSP32 concentrations under basal conditions, resulting in a lesser heat shock response when those persons are subjected to 1 h of exertional-heat stress. Elevated levels of TLR4 and NF-κB were also shown at baseline following NZBC-extract supplementation, but the overall impact of these changes is questionable because in our prior work (Lee et al. 2022) we did not detect any differences between study conditions in IL-1RA, IL-6, or IL-10.

### Supplementary Information

Below is the link to the electronic supplementary material.

Supplementary file1 (DOCX 21 KB)

## Data Availability

Depending on the rationale and scenario, the reader could email the corresponding author for access to study data.
