# Supplementary material for: New Zealand blackcurrant extract modulates the heat shock response in men during exercise in hot ambient conditions
Source: Eur J Appl Physiol. 2024 Mar 7;124(8):2315–28. doi: 10.1007/s00421-024-05439-w (PMC11322260; doi:10.1007/s00421-024-05439-w)
Supplement: Supplementary file 1 — Supplementary file1 (DOCX 21 KB) [file 421_2024_5439_MOESM1_ESM.docx]

Supplemental Table 1. Summary of Primary Antibodies Used for Western Blot Analysis

| **Primary Antibody Name** | **Antibody Distributor** | **Antibody Identifier** | **Product Number** |
| --- | --- | --- | --- |
| B-cell lymphoma protein | Santa Cruz Biotechology | BCL-2 (C-2) | sc-7382 |
| BCL-2 associated X, apoptosis regulator | Santa Cruz Biotechology | BAX (2D2) | sc-20067 |
| Beta Actin | Santa Cruz Biotechology | β-Actin (C4) | sc-47778 |
| Caspase-9 p10 | Santa Cruz Biotechology | Caspase-9 p10 (F-7) | sc-271759 |
| Heat shock protein 32 | Santa Cruz Biotechology | HO1 (H-105) | sc-10789 |
| Heat shock protein 60 | Santa Cruz Biotechology | HSP 60 (N-20) | sc-1052 |
| Heat shock protein 72 | Santa Cruz Biotechology | HSP 70/HSC 70 (W27) | sc-24 |
| Heat shock protein 90 | Enzo | HSP90α | sps-771-D |
| Myeloid Differentiation Factor 88 | Santa Cruz Biotechology | MyD88 (E-11) | sc-74532 |
| Nuclear factor kappa beta | Abcam | NFKB p65 | ab16502 |
| Phosphorylated 5’-AMP-activated protein kinase | Santa Cruz Biotechology | pAMPKα1 (Ser 496): | sc-101631 |
| Phosphorylated inhibitor of kappa beta | Abcam | p-IκB-α (S36) | ab133462 |
| Toll like receptor 4 | Santa Cruz Biotechology | TLR4 (25) | sc-293072 |
